# Premedication Does Not Influence the Incidence of Infliximab Infusion Reactions in Pediatric Patients with Inflammatory Bowel Disease—A Single Center Case–Control Study

**DOI:** 10.3390/jcm10143177

**Published:** 2021-07-19

**Authors:** Edyta Szymanska, Maciej Dadalski, Joanna Sieczkowska-Golub, Dorota Jarzebicka, Monika Meglicka, Marcin Osiecki, Anna Wiernicka, Dariusz M. Lebensztejn, Bartosz Korczowski, Jaroslaw Kierkus

**Affiliations:** 1 Department of Gastroenterology, Hepatology, Feeding Disorders and Pediatrics, The Childrens’ Memorial Health Institute, 04-730 Warsaw, Poland; m.dadalaski@ipczd.pl (M.D.); j.sieczkowska@ipczd.pl (J.S.-G.); d.jarzebicka@ipczd.pl (D.J.); m.meglicka@ipczd.pl (M.M.); n.osiecki@ipczd.pl (M.O.); a.wiernicka@ipczd.pl (A.W.); j.kierkus@ipczd.pl (J.K.); 2 Department of Pediatrics, Gastroenterology, Hepatology, Nutrition and Allergology, Medical University of Bialystok, 15-089 Białystok, Poland; lebensztejn@hoga.pl; 3 Department of Pediatrics and Pediatric Gastroenterology, College of Medical Sciences, University of Rzeszów, 35-310 Rzeszów, Poland; korczowski@op.pl

**Keywords:** premedication, infusion reaction, infliximab, inflammatory bowel disease

## Abstract

Background: Infusion reactions (IRs) are the most common adverse events (AEs) of infliximab (IFX) treatment in patients with inflammatory bowel disease (IBD). Prophylactic premedication (PM) with corticosteroids or antihistamines prior to IFX infusions has been used in clinical practice, but its efficacy is not known. The aim of this study was to assess the influence of steroid PM on IR incidence in pediatric patients with IBD receiving IFX. Methods: We performed a case–control study that included pediatric patients with IBD receiving IFX. Patients were divided into four subgroups according to the agent and PM they received: Remicade (original drug) + PM, and two biosimilars—Reshma +/− PM, and Flixabi—PM. At our site, until 2018, PM with steroids was used as a part of standard IFX infusion (PM+); however, since then, this method has no longer been administered (PM−). IRs were divided into mild/severe reactions. Differences between subgroups were assessed with the appropriate chi-square test. Multivariate logistic regression was used to assess associations between PM and IR incidence, correcting for co-medication usage. Results: There were 105 children (55 PM+, 44 male, mean age 15 years) included in the study who received 1276 infusions. There was no difference between the PM+ and PM− subgroups, either in incidence of IR (18.2% vs. 16.0% of patients, *p* > 0.05) or in percentage of infusions followed by IR (2.02% vs. 1.02% of infusions, *p* > 0.5). The OR of developing IR when using PM was 0.34, and the difference in IRs ratio in PM+ and PM− patients was not statistically significant (95% CI, 0.034–1.9). There were 11/18 (61.1%) severe IRs (anaphylactic shock) reported in all patients (both PM+ and PM−). Conclusion: At our site, the incidence of IR was low, and PM did not decrease the incidence of IR in pediatric patients with IBD receiving IFX. These results indicate that PM with steroids should not be a standard part of IFX infusion to prevent IR.

## 1. Introduction

Infliximab (IFX) is a chimeric monoclonal antibody. It works by binding to TNF-α, a cytokine that plays a key role in the autoimmune reaction. In chronic inflammatory diseases (e.g., rheumatoid arthritis, inflammatory bowel disease—IBD) IFX seems to work by preventing TNF-α from binding to its receptor in the cell, which blocks further cytokine inflammatory cascade and leads to the development of disease [1].

Biologic treatment with IFX has changed the management patterns of IBD with better efficacy, lowering the rate of surgeries and other healthcare resources related to complications or worsening of the disease [2,3].

Remicade, the first IFX agent, has been widely used in induction and maintenance of remission in patients with IBD for over 15 years. Its safety and efficacy have been well proven [4,5].

Since 2014, in Poland, “so-called” biosimilar drugs (e.g., Remsima, Inflectra, Flixabi) that are Remicade generics are also available on the market. Thus far, there are enough data confirming their safety and efficacy comparable to the original agent [6,7].

Biologics are generally well-tolerated and safe. One of the most common adverse events (AEs) associated with IFX administration are infusion reactions (IRs). Published data have demonstrated that up to 25% of patients treated with IFX experience hypersensitivity reactions [8]. An overall incidence of both acute and delayed IRs in patients with Crohn’s disease (CD) reported in hospitals was 6.1%, occurring in 9.7% of all patients, and severe acute reactions accounted for 1.0% of all infusions [9]. Long-term registry and retrospective studies demonstrated a lower per-infusion incidence: 1.3–3.7% of all infusions [10,11]. It is assumed that infusion incidence may be higher in short-term studies due to the high frequency of acute reactions occurring during initial infusions [12].

Moreover, children may have a different immune response to biological agents than adults or young adult patients. Since their immune system is still developing, they may be more prone to developing IRs [13].

Prophylactic premedication (PM) with corticosteroids and/or antihistamines prior to IFX infusion is widely used in clinical practice but its efficacy is questionable due to a lack of pathophysiological rationale and verification by controlled trials [14].

The aim of this study was to investigate the influence of PM on incidence of IRs in children IBD patients receiving IFX.

## 2. Methods

We performed a retrospective case–control study in one reference hospital that included pediatric patients with IBD receiving IFX. The analyzed data from patients’ medical charts included the period from 2013 until May 2020. Children were divided into 4 subgroups according to both the agent they received and whether PM prior to IFX infusion was administered: Remicade (original drug) + PM, and 2 biosimilars—Remsima +/− PM, and Flixabi −PM, respectively.

At our site, until 2018, PM with steroids (corhydrone/hydrocortisone) was used as a part of standard IFX infusion (PM+); however, since then, this method has no longer been administered (PM−). In the case of Remsima, from 2014 until 2018, all patients received PM, then the PM was completed; none of them have received PM since 2018.

IRs were divided into mild (skin changes)/severe reactions (anaphylactic shock).

When the IR was mild, patients did not complete the medication—the PM was administered before an injection but, in case of anaphylactic shock, the drug was stopped.

Statistical analysis was performed using Statisctica 12.0 (StatSoft, Krakow, Poland). Standard descriptive statistical analyses were performed, including frequency distributions for categorical data and calculation of median and interquartile range (IQR) for continuous variables.

Differences between subgroups were assessed with the appropriate chi-square test. Multivariate logistic regression was used to assess associations between PM and IR incidence, correcting for co-medication usage.

## 3. Results

There were 105 children (55 PM+, 44 male, mean age 15 years) included in the study who received 1276 infusions; 720 PM+ and 556 PM−. In our group, a majority of patients (70/105–66.7%) were treated with Remsima as a biologic agent. Ten of fifty-five PM+ patients (18.2%) developed IFX IRs that comprised: anaphylactic shock (four patients), urticaria (three patients), and rush/erythema (three patients). Eight of fifty PM− children had the following IRs: anaphylactic shock (seven patients) and erythema (one patient). All IRs resolved after steroid/antihistamine or adrenaline administration, and patients with mild IRs (skin changes) could proceed with their IFX therapy. Only one patient who had IFX commenced with PM, and then, after some time, continued without it, developed an anaphylactic reaction once the steroid was not administered prior to infusion.

Table 1a,b presents patients’ characteristics.

**Figure 1:**
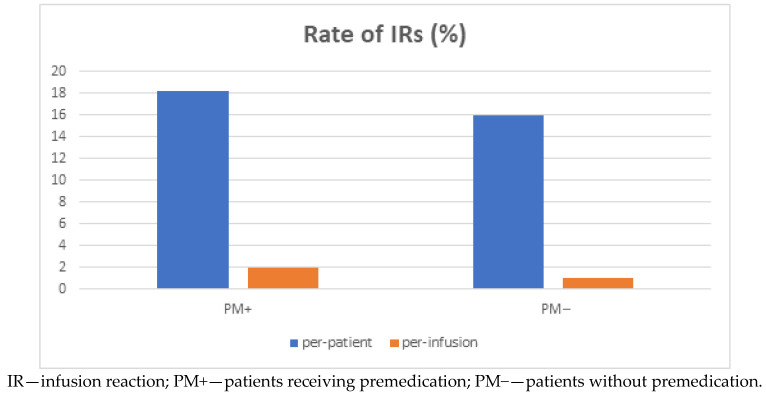
Incidence rate (%) of IRs per patient and per infusion according to premedication administration.

There was no difference between the PM+ and PM- subgroups in either incidence of IR (18.2% vs. 16.0% of patients, *p* > 0.05) or percentage of infusions followed by IR (2.02% vs. 1.02% of infusions, *p* > 0.5) (Figure 1). Patients who started their biologic treatment with obligatory PM prior to IFX infusion and continued with the therapy over the time when it was no longer routinely provided did not develop anymore IRs without PM.

Data concerning number of infusions until developing IRs are not available for all patients; however, among the three patients who received PM, IRs appeared at first dose, as well as after the second and fourth doses, respectively. For seven patients who did not receive PM, an average number of infusions until the development IRs was eight, i.e., from 2 to 16 doses.

The OR of developing IR when using PM was 0.34, and without PM it was 0.54. The difference in IR ratio in PM+ and PM− patients was not statistically significant (95% CI, 0.034–1.9).

## 4. Discussion

Published data and our experience indicate that IRs to IFX have a low rate of occurrence. It applies to both the per-infusion reaction rate as well as the per-patient reaction rate. Moreover, the majority of acute IRs are mild or moderate in severity and resolve with rate adjustments or administration of antihistamines or steroids [15].

However, the immunomodulatory properties of biologics have demanded careful evaluation of their safety profiles. At the beginning, IFX has been administered either under a physician’s supervision or following PM with steroids and/or antihistamines due to concerns over the development of AEs, including IRs [16].

At our site, from 2003 until 2018, obligatory PM (most often with corhydrone) was administered prior to IFX infusion. We have reported 10 IRs per 55 PM+ patients, which accounts for 18.2% of patients receiving IFX, and it was comparable to the rate of IRs (16%) noted when PM was no longer provided as obligatory (since February 2018) [17].

Eleven IRs were anaphylactic shock, and all of them resolved after steroid/antihistamine or adrenaline administration. Our results are comparable to the observations of other authors [18,19].

In the TREAT registry, a multicenter, prospective, observational registry of CD patients in the USA, the per-infusion reaction rate to IFX was 3.0%, with 0.05% of infusions classified as serious (53,003 total infusions in 3322 patients) [20].

A systemic review and meta-analysis by Fumery et al. [8] including three IBD studies (and seven of other immune-mediated inflammatory diseases) has demonstrated that neither steroid PM nor antihistamines administration prior to IFX infusion was associated with a decreased risk of IRs. Moreover, the combination of corticosteroids and antihistamines did not decrease the risk of IRs either [8].Our experience and results from this study confirm this observation.

Jacobstein et al. [16] studied the proportion of pediatric patients with IBD receiving IFX who developed IRs and the potential effects of PM on IR. A total of 1652 infusions given to 243 patients in 6 centers was analyzed, and IRs occurred in a small proportion of infusions among these patients. Premedication did not seem to prevent the development of IRs. However, the authors observed that once an IR had occurred, PM might be indicated to prevent subsequent IRs [16]. We had the same experience. There were patients in our group who received PM prior to IFX when PM was obligatory, and they continued with biologic therapy over the time when it was no longer routinely provided. Therefore, we could observe the potential effects of PM on IRs—their rate was comparable with or without PM. Moreover, in mild hypersensitivity reactions, the administration of steroid/antihistamines usually prevents subsequent IRs, meaning that therapy could proceed. Only one patient who had IFX commenced with PM; then, after some time, they proceeded without it and developed an anaphylactic reaction once the steroid was not administered prior to infusion.

A study from the Netherlands investigating the influence of steroid PM on incidence of IR in 226 children with IBD receiving 3433 infusions of IFX also confirmed that the incidence of IR is low, and PM does not decrease the incidence of IRs in these patients [15].

Comparing the results from adults and pediatric studies, both of them show low and rather comparable rates of IR. However, we have not found any study that compares these age groups, which would be interesting and clinically important.

Another important issue related to the safety profile of IFX concerns biosimilars. The widespread availability of the cheaper, generic versions of originator was associated with many fears from both patients and physicians. Currently, there are enough data confirming their safety and efficacy as being comparable to original IFX [6,7,21].

Our study is the only one that not only investigates IRs’ rate with or/without PM but that also compares original IFX with two biosimilars (Remsima, Flixabi). Due to the even greater potential immunomodulatory properties of biosimilar agents, there were concerns whether their use would not be associated with an increased rate of IRs. We have analyzed four groups of patients: those treated with IFX originator (Remicade) and PM; those receiving Remsima both with and without PM; and those on Flixabi without PM. The IR rate was comparable between the originator and biosimilars, and PM had no statistically significant influence on this rate for all the agents.

The limitation of our study is its retrospective character and, as the numbers of IRs, are limited, some differences might not appear.

Results from our study and the available published data indicate that PM either with steroids or antihistamines does not seem to be associated with a decreased risk of IRs to IFX in IBD patients. Therefore, we assume that PM should no longer be a part of standard protocols.

## 5. Conclusions

At our site, the incidence of IR was low, and PM did not decrease the incidence of IR in Polish pediatric patients with IBD receiving IFX. These results indicate that PM with steroids and/or antihistamines should not be a standard part of IFX infusion to prevent IR.

## Figures and Tables

**Table 1:** (**a**) Characteristics of pediatric patients with IBD receiving IFX. (**b**) General data for all patients.

(a)				
	Remicade	Remsima	Flixabi	All Patients
No of patients	19	70	16	105
PM+	19	36	0	55
PM−	0	34	16	50
IR	8	2	8	18
-anaphylactic shock	3	1	7	11
-urtricaria	2	1	0	3
-erythema/rush	3	1	1	5
**(b)**				
Male	44 (41.9%)			
Age range yr.	15 (6–18)			
No of infusions	1276			
CD	91 (86.7%)			

No—number; yr.—year; CD—Crohn’s disease; PM—premedication; IR—infusion reaction.

## Data Availability

Data is contained within the article.

## References

[1] Guo Y., Lu N., Bai A. (2013). Clinical use and mechanisms of infliximab treatment on inflammatory bowel disease: A recent update. Biomed. Res. Int..

[2] Hyams J., Crandall W., Kugathasan S., Griffiths A., Olson A., Johanns J. (2007). Induction and maintenance infliximab therapy for the treatment of moderate-to-severe Crohn’s disease in children. Gastroenterology.

[3] Hyams J.S., Lerer T., Griffiths A., Pfefferkorn M., Kugathasan S., Evans J., Markowitz J. (2009). Long-term outcome of maintenance infliximab therapy in children with Crohn’s disease. Inflamm. Bowel Dis..

[4] Lichtenstein G.R., Feagan B.G., Cohen R.D., Salzberg B.A., Safdi M., Popp J.W., Langholff W., Sandborn W.J. (2018). Infliximab for Crohn’s Disease: More Than 13 Years of Real-world Experience. Inflamm. Bowel Dis..

[5] Corica D., Romano C. (2017). Biological Therapy in Pediatric Inflammatory Bowel Disease: A Systematic Review. J. Clin. Gastroenterol..

[6] Armuzzi A., Fiorino G., Variola A., Manetti N., Fries W., Orlando A., Maconi G., Bossa F., Cappello M., Biancone L. (2018). The PROSIT Cohort of Infliximab Biosimilar in IBD: A Prolonged Follow-up on the Effectiveness and Safety Across Italy. Inflamm. Bowel Dis..

[7] Kim N.H., Lee J.H., Hong S.N., Yoon H., Kang H.W., Lee S.H., Park D.I. (2019). Long-term Efficacy and Safety of CT-P13, a Biosimilar of Infliximab, in Patients with In-flammatory Bowel Disease: A Retrospective Multicenter Study. J. Gastroenterol. Hepatol..

[8] Fumery M., Tilmant M., Yzet C., Brazier F., Loreau J., Turpin J., Le Mouel J.P., Goeb V., Nguyen-Khac E., Singh S. (2019). Premedication as primary prophylaxis does not influence the risk of acute infliximab infusion reactions in immune-mediated inflammatory diseases: A systematic review and meta-analysis. Dig. Liver Dis..

[9] Cheifetz A., Smedley M., Martin S., Reiter M., Leone G., Mayer L., Plevy S. (2003). The incidence and man- agement of infusion reactions to infliximab: A large center experience. Am. J. Gastroenterol..

[10] Ducharme J., Pelletier C., Zacharias R. (2010). The safety of inflixi- mab infusions in the community setting. Can. J. Gastroenterol..

[11] Choquette D., Faraawi R., Chow A., Rodrigues J., Bensen W.J., Nantel F. (2015). Incidence and management of infusion reactions to infliximab in a prospective real-world community registry. J. Rheumatol..

[12] Lichtenstein L., Ron Y., Kivity S., Ben-Horin S., Israeli E., Fraser G.M., Dotan I., Chowers Y., Confino-Cohen R., Weiss B. (2015). Infliximab-Related Infusion Reactions: Systematic Review. J. Crohn’s Colitis.

[13] Kaur B.P., Secord E. (2019). Innate Immunity. Pediatr. Clin. N. Am..

[14] Picoraro J., Winberry G., Siegel C.A., El-Matary W., Moses J., Grossman A., Park K.T. (2017). Premedication Use Be-fore Infliximab Administration: A Cross-sectional Analysis. Inflamm. Bowel Dis..

[15] Checkley L.A., Kristofek L., Kile S., Bolgar W. (2018). Incidence and Management of Infusion Reactions to Infliximab in an Alternate Care Setting. Dig. Dis. Sci..

[16] Kostev K., Bauer A., Jacob L. (2019). Lines of therapy with biological drugs in dermatology, gastroenterology, and rheumatology practices in Germany. Int. J. Clin. Pharmacol. Ther..

[17] Szymanska E., Dadalski M., Oracz G., Kierkus J. (2015). Safety profile of biologic therapy in Polish paediatric patients with Crohn’s disease. Gastroenterol. Rev..

[18] Van Wassenaer E.A., Meester V.L., Kindermann A., Koot B.G., Benninga M.A., de Meij T.G. (2019). Premedication with Intravenous Steroids Does Not Influence the Incidence of Infusion Reactions Following Infliximab Infusions in Pediatric Inflammatory Bowel Disease Patients-A Case-Control Study. Eur. J. Clin. Pharmacol..

[19] Jacobstein D.A., Markowitz J.E., Kirschner B.S., Ferry G., Cohen S.A., Gold B.D., Baldassano R.N. (2005). Premedication and Infusion Reactions with Infliximab: Results from a Pediatric Inflammatory Bowel Disease Consortium. Inflamm. Bowel Dis..

[20] Lichtenstein G.R., Feagan B.G., Cohen R.D., Salzberg B.A., Diamond R.H., Price S., Langholff W., Londhe A., Sandborn W.J. (2012). Serious Infection and Mortality in Patients with Crohn’s Disease: More Than 5 Years of Follow-Up in the TREAT™ Registry. Am. J. Gastroenterol..

[21] Sieczkowska-Golub J., Meglicka M., Plocek A., Banaszkiewicz A., Jarzebicka D., Toporowska-Kowalska E., Gawrońska A., Oracz G., Kierkus J. (2017). Induction Therapy with Biosimilar Infliximab in Children with Crohn Disease. J. Pediatr. Gastroenterol. Nutr..

